# A Rat Model of Central Venous Catheter to Study Establishment of Long-Term Bacterial Biofilm and Related Acute and Chronic Infections

**DOI:** 10.1371/journal.pone.0037281

**Published:** 2012-05-16

**Authors:** Ashwini Chauhan, David Lebeaux, Benoit Decante, Irene Kriegel, Marie-Christine Escande, Jean-Marc Ghigo, Christophe Beloin

**Affiliations:** 1 Institut Pasteur, Unité de Génétique des Biofilms, Département de Microbiologie, Paris, France; 2 Laboratoire de Recherche Chirurgicale, Centre Chirurgical Marie Lannelongue, Le Plessis Robinson, France; 3 Service Anesthésie-Réanimation-Douleur, Hôpital de l'Institut Curie, Paris, France; 4 Laboratoire de Microbiologie Médicale, Hôpital de l'Institut Curie, Paris, France; Université d'Auvergne Clermont 1, France

## Abstract

Formation of resilient biofilms on medical devices colonized by pathogenic microorganisms is a major cause of health-care associated infection. While *in vitro* biofilm analyses led to promising anti-biofilm approaches, little is known about their translation to *in vivo* situations and on host contribution to the *in vivo* dynamics of infections on medical devices. Here we have developed an *in vivo* model of long-term bacterial biofilm infections in a pediatric totally implantable venous access port (TIVAP) surgically placed in adult rats. Using non-invasive and quantitative bioluminescence, we studied TIVAP contamination by clinically relevant pathogens, *Escherichia coli*, *Pseudomonas aeruginosa*, *Staphylococcus aureus* and *Staphylococcus epidermidis*, and we demonstrated that TIVAP bacterial populations display typical biofilm phenotypes. In our study, we showed that immunocompetent rats were able to control the colonization and clear the bloodstream infection except for up to 30% that suffered systemic infection and death whereas none of the immunosuppressed rats survived the infection. Besides, we mimicked some clinically relevant TIVAP associated complications such as port-pocket infection and hematogenous route of colonization. Finally, by assessing an optimized antibiotic lock therapy, we established that our *in vivo* model enables to assess innovative therapeutic strategies against bacterial biofilm infections.

## Introduction

Microbial contamination originating from patients and health-care workers leads to formation of bacterial and fungal biofilms on the surface of indwelling medical devices [1], [2]. In addition to mechanical hindrance, device-associated biofilms are a primary cause of hospital-acquired (nosocomial) infections that are difficult to eradicate due to the high tolerance of biofilms towards antimicrobial and host defenses [3], [4]. Currently, although biofilms constitute a major threat to patients, there is no truly efficient method for eliminating biofilms except the traumatic removal of contaminated devices [5], [6]. These invasive procedures often involve difficult therapeutic decisions leading to increased cost of additional treatments [7], [8]. Hence, in light of this socio-economic burden, there is a need for efficient prophylaxis against pathogenic biofilm developing on implanted medical devices.

A number of anti-biofilm strategies have recently emerged from *in vitro* studies of the biofilm physiology of model bacteria [9], [10], [11], [12]. Moreover several animal models using subcutaneously implanted or vascular catheters were successfully used to evaluate the antimicrobial efficacy of several antibiotics against bacteria such as *Staphylococcus aureus* and *Staphylococcus epidermidis*, and to assess the role of regulators or virulence factors in biofilm development [9], [13], [14], [15], [16], [17], [18], [19]. However, these approaches were mainly designed to study short-term biofilm infections, and rarely integrated both microbial and host contribution into development of biofilm infections.

Repeated access to totally implantable venous access ports (TIVAP) often leads to formation of intraluminal biofilms which constitute reservoirs of local and systemic infections in human patients [20]. In order to study catheter-related biofilm infections, we developed an *in vivo* model of long-term biofilm infection using pediatric TIVAP surgically implanted in adult rats. We used immunocompetent and immunosuppressed rats to monitor TIVAP contamination by clinically relevant bioluminescent pathogens, including *Escherichia coli*, *Pseudomonas aeruginosa*, *S. aureus and S. epidermidis*. We show that i) the bacterial population colonizing TIVAP display all classical biofilm phenotypes and ii) that the immunological status of implanted rats determine the onset of bloodstream infection and organ colonization upon release of bacteria from TIVAP biofilms. Finally, we show that our model reproduces most clinically relevant situations associated with TIVAP-related infections and we demonstrate its potential in evaluating the efficiency of biofilm antibiotic lock eradication strategies.

This model contributes to a better understanding of device-related infections and constitutes a relevant approach for validating diagnostic and therapeutic anti-biofilm approaches.

## Materials and Methods

### Microbiological evaluation of extracted TIVAP

#### Microbiological analysis procedure

TIVAP catheter tip culture was performed on blood agar by a quantitative method with a 10^3^ colony-forming unit (CFU)/mL threshold, as described [6], [21]. Species identification of *Enterobacteriaceae*, coagulase-negative staphylococci and yeasts relied on use of API® 20E, API® Staph and API ® 20C AUX test kits (BioMérieux, Marcy l'Etoile, France), respectively. Those data were generated as part of routine activities in the clinical microbiology laboratory of the Institut Curie hospital. Between 2007 and 2010, 600 TIVAP were removed for suspicion of infection and 279 cultures (46.5%) were positive with at least one microorganism. Those TIVAP originated mostly from adult patients (219/279, 78%) with a median age of 43 [0–83] years. Finally, 292 microorganisms were responsible for those 279 TIVAP-related infections (Table S1).

### Bacterial strains

Luminescent variants of four clinically relevant pathogens, i.e. *E. coli, P. aeruginosa*, *S. aureus* and *S. epidermidis* were purchased (*S. aureus* Xen36 and *S. epidermidis* Xen43 from Caliper) or gifted (*P. aeruginosa* Lm1, a bioluminescent derivative of the PAK clinical strain [22] and *E. coli* EAEC 55989 [23] transformed with stable plasmid pAT881 [24]). *E. coli* and *P. aeruginosa* strains were grown in lysogeny broth (LB); *S. aureus* Xen36 and *S. epidermidis* Xen43 were cultured in tryptic soy broth (TSB) supplemented with 0.2% glucose at 37°C.

### *In vitro* biofilm formation

**Figure 1:**
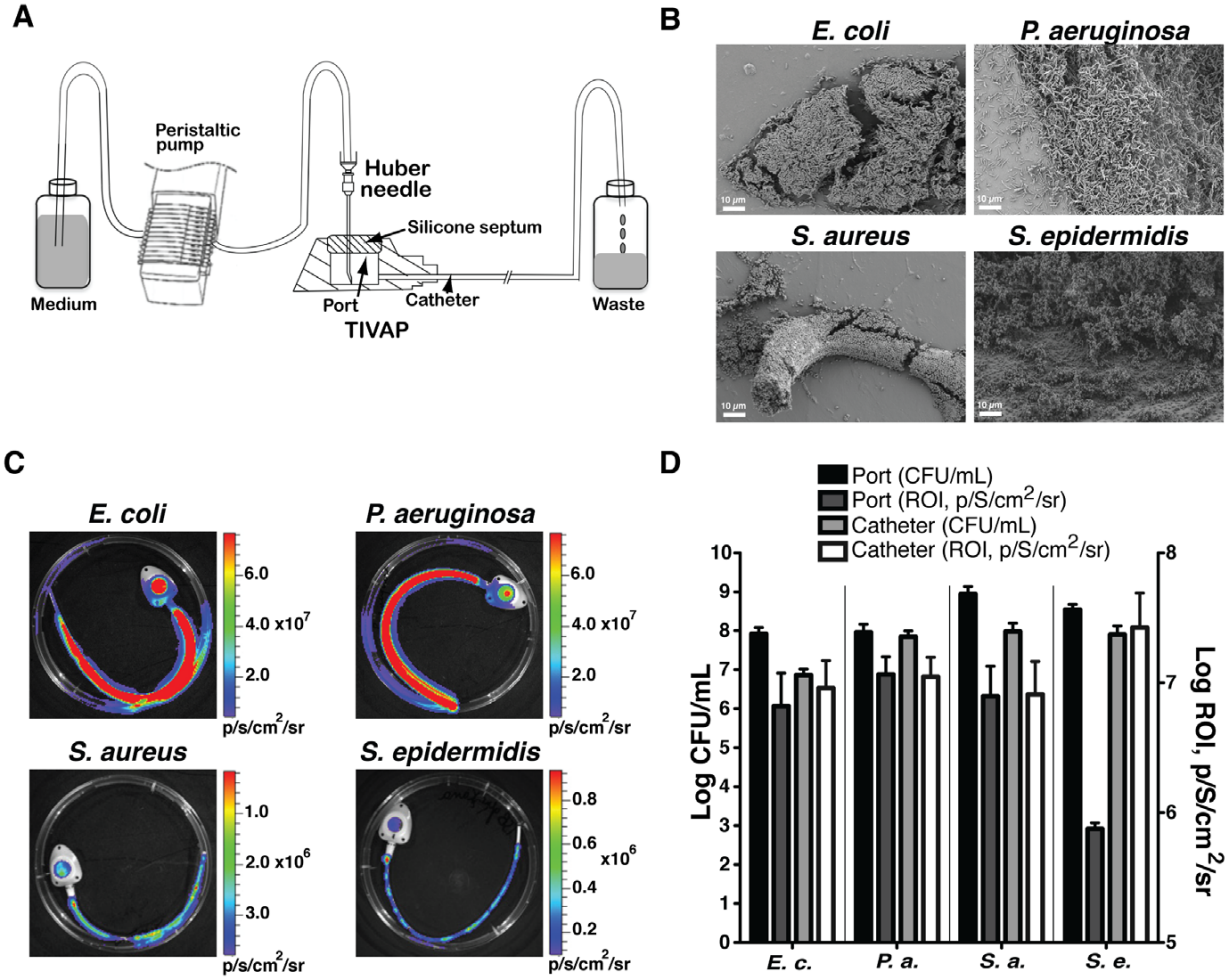
Monitoring of *in vitro* biofilm-forming capacity of clinically relevant strains. (A) *in vitro* continuous flow system to grow biofilms inside TIVAP. After 24 h biofilm formation in TIVAP was confirmed by scanning electron microscopy (SEM) for the four bacteria (B) and bioluminescence activity was acquired using an IVIS 100 camera (C). (D) Biofilm was analyzed by plating CFU/mL (n = 3 for each bacteria) on LB agar (E.c., *E. coli* or P.a., *P. aeruginosa*) or TSB agar (S.a., *S. aureus* or S.e., *S. epidermidis*) plates. Relative luminescence was correlated with CFU/mL by quantitative analysis of luminescence signals in the port and catheters separately using Living Image software.

All experiments were carried out at least in triplicate. Catheter-associated biofilms were developed in the lumen of pediatric TIVAP (cat # 2105ISP, Polysite Micro, Titanium 2000 series, Perouse Medical, France) composed of a titanium microport with an 8 mm silicone septum and a radio-opaque catheter with 0.65 mm internal diameter. A Huber needle was inserted into the silicone septum of the port for continuously supplying fresh medium from reservoir bottles (Figure 1). The system is set up in laminar airflow to maintain sterility. A bacterial dose of 100 cells/100 µL was optimal to form intraluminal biofilms in TIVAP *in vitro*. Bacterial inoculum of 100 cells/100 µL was injected through the septum of the port and allowed to adhere to the TIVAP surface for 3 h at 37°C before continuous flow was turned on at a speed of 300 µL/min for 24 h. The spent media and non-adherent cells were collected in a waste container. Control catheters were prepared without bacterial inoculation. TIVAP were disconnected under sterile conditions and monitored for bioluminescence using an IVIS-100 imaging system with a charge-coupled device (CCD) camera (Xenogen Corporation, Alameda, CA, USA). The CFU/mL and bioluminescent signal (ROI, p/S/cm^2^/sr) correlation was determined by separately comparing viable cell counts obtained for catheter and port.

### Extraction and quantification of biofilm bacteria

TIVAP were carefully wiped with 70% EtOH before extracting intraluminal biofilm bacteria to avoid contaminant. The catheter was cut into small pieces and a slit was made horizontally to expose the lumen and transferred to a tube containing 1 ml sterile 1X phosphate-buffered saline (PBS). The septum was removed from the port using a sterile scalpel and forceps, cut into small pieces and transferred to a separate tube containing 1 mL sterile 1X PBS. Cells attached to the titanium body of the port were scratched in 100 µL 1X PBS and transferred to the same tube as the septum. Biofilm that formed on the septum and in the lumen of catheter was extracted by vigorously vortexing the tubes for 1 min, followed by transferring them to an ultrasonic water bath (NEYtech Ultrasonik, 44–48 Khz) for 5 min. The bacterial suspension was then diluted serially, plated on agar plates and incubated at 37°C for colony counts. CFU/mL and bioluminescent signals (ROI, p/S/cm^2^/sr) were plotted together for correlation.

### Animal model

#### Catheter placement

CD/SD (IGS∶Crl) male rats (Charles River) weighed 275–300 g and were acclimatized to a 12 h day/night cycle for one week prior to use. Animals were anesthetized using a 500 µL cocktail (7∶2∶1, v/v) of ketamine (Imalgen 1000, Merial SAS Lyon, FR), xylazine (Rompun 2%, Bayer Healthcare, Germany) and acepromazine (Calmivet Solution Injectable, Vetoquinol SA, Lure, FR) given intraperitoneally. Rats were shaved prior to placement of TIVAP and their skin was cleaned using 70% alcohol and 10% povidone-iodine solution.

#### Surgical placement of TIVAP

The port was implanted at dorsal midline towards the lower end of the thoracic vertebrae by creating a subcutaneous pocket. The port was held in place using sutures. 10/11 cm catheter was tunneled subcutaneously using a tunneling rod onto the ventral side in the clavicle region, cut open by an incision to expose the right external jugular vein for catheter insertion. The catheter was inserted into the jugular vein by making a micro-incision and progressively moved into the superior vena cava up to the right atrium. The catheter was held in place by ligating proximally and distally with sterile surgical silk thread (Ethicon, 3-0). Functionality of TIVAP was maintained by flushing 1X sterile PBS followed by a heparin lock (500 IU/mL) every day. Prior to inoculation of clinical strains, all rats were checked for the absence of infection by plating 100 µL blood as well as monitoring rats for the absence of luminescence signals.

#### TIVAP contamination in immunocompetent rats

The inoculum dose was optimized by injecting within TIVAP increasing doses of each bacteria and then selecting the smaller inoculum giving an observable bioluminescent signal as compared to TIVAP injected with PBS 1X. The inoculum dose was optimized to 10^4^ cells for *E. coli*, 10^6^ cells for *P. aeruginosa* and *S. aureus*. For *S. epidermidis* the maximum dose that could be used was 10^8^ cells but did not lead to bioluminescent-detectable colonization (see Results). Overnight grown cultures were diluted in 1X PBS to the optimized inoculum dose. The inoculum dose was also confirmed by plating it for CFU/mL on respective antibiotic-containing plates. Briefly, 100 µL 1X PBS containing the optimized inoculum cell size was injected through a silicone septum into the port using a Huber needle (total TIVAP dead volume 250 µL). Control rats received 1X PBS. Progression of colonization from the port to the distal end of the catheter was monitored using an IVIS100 imaging system.

### Immunosuppression and infection in catheterized rats

Rats were injected intraperitoneally with cyclophosphamide (Sigma Aldrich cat# C0768-5G). Dose and regimen of cyclophosphamide delivery were optimized by estimating blood total leukocyte count as determined using an animal blood cell counter (Vet abc, SCIL, Germany). Furthermore, the physical state of rats such as lack of appetite, reduced weight and loss of hair indicated immunosuppressed status. One hundred mg/kg body weight of cyclophosphamide was finally selected for giving intraperitoneal injections to rats (n = 4 for each bacterial strain) on day -4 and 50 mg/kg on day -1 of inoculation. An optimized inoculum dose of 10^2^ cells/100 µL 1X PBS for all bacterial strains (higher inoculum doses lead to the death of animals overnight) was used for TIVAP contamination and was confirmed by plating for CFU/mL. Control catheterized and immunosuppressed rats received 100 µL 1X PBS only. Prior to inoculation of clinical strains, all rats were checked for the absence of infections as for immunocompetent rats.

### Peripheral blood and organ sampling

Blood was aseptically sampled (at day 4 and day 8 for immunocompetent rats and day 3 for immunosuppressed rats) by puncturing the retro-orbital sinus plexus of anesthetized rats with a Pasteur pipette. One hundred and fifty microliters of blood was transferred to a tube containing 10 µL of 100 IU/mL heparin (day 4 and day 8) whereas another 50 µL was transferred to an eppendorf tube containing 50 µL 0.01 M EDTA stored on ice until analyzed (day 4 post infection). Blood with heparin was serially diluted and plated on antibiotic plates for CFU/mL estimation. Blood in EDTA was used for TLC (total leukocyte count) and DLC (differential leukocyte count) using automated blood cell analyzer (ABC vet 2.0, Germany) post day 4 of infection. Metastatic infection resulting from TIVAP-associated biofilm was determined by aseptically harvesting organs (heart, lungs, liver and kidneys) on the final day of the experiment. Organs were weighed and homogenized in 5 mL 1X sterile PBS and serial dilutions were plated for estimating the CFU/organ.

### Electron microscopy

After aseptic removal of colonized TIVAP from rats, 1 cm catheter of the catheter tip was cut and the septum was dissected from the port using a scalpel. Septum and catheter pieces were washed twice in cacodylate solution (0.07 M) and then fixed in EM fixative solution. Samples were stored at 4°C until sent for microscopy (not more than 10 days).

### Hematogenous infections

*S. aureus* Xen36 was used to check the possibility of TIVAP colonization through the venous system. An inoculum of 5×10^8^ cells of *S. aureus* Xen36 was centrifuged, washed and resuspended in 500 µL 1X PBS. Bacteria were injected into the bloodstream through the lateral tail vein. Animals were monitored for luminescence using the IVIS-100 imaging system for infection and colonization. The animals were sacrificed on day 3 after injection and TIVAP were removed to confirm colonization by luminescence and plating CFU/mL.

### *In vivo* antibiotic lock technique (ALT/antibiotic lock therapy)

Antibiotics and their concentration for ALT studies were chosen based on recommendations made by IDSA guidelines [6]. Vancomycin, cefazolin and gentamicin were obtained from Sigma Aldrich. The minimum inhibitory concentration (MIC) of all drugs used in the study was determined by the macrodilution method in Eppendorf tubes against all bacterial strains used in the study. Cefazolin, Gentamicin and Vancomycin MICs of *S. aureus* Xen36 were 0.625, 0.3125 and 1 µg/mL respectively. Efficacy of cefazolin ALT (5,000 µg/mL, 5000 IU/mL heparin) and gentamicin ALT (1,000 µg/mL, 2,500 IU/mL heparin) was evaluated against *S. aureus* Xen36. The 4-day-old biofilm formed inside the implanted TIVAP was locked (200 µL) with the above-discussed antibiotics and monitored for biofilm clearance by measuring luminescence. The lock was replaced every 24 h and the ALT regimen (n = 4 rats for each treatment) was followed for 5 days along with daily subcutaneous injection of vancomycin (50 mg/Kg), except for the first set of experiments where a significant mortality (∼30%, n = 3/9) occurred following ALT regimen without systemic treatment. Therefore, it was necessary to treat the animal with systemic vancomycin during the course of ALT treament. Rats were sacrificed after day 5 for estimating viable cell count. For evaluating the efficacy of the complex ALT solution against *S. aureus* Xen36 biofilm, highly concentrated doses of gentamicin and cefazolin were mixed in a final 1∶1 (v/v) ratio and used to lock colonized TIVAP. Untreated and colonized implanted TIVAP were used as controls.

### Ethics Statement

#### Clinical sampling

Extracted TIVAP are considered medical wastes by the Integrated Clinical Research Center of the Institut Pasteur. The project consisting of analysis of the bacterial flora of TIVAP was registered under the number 2009-44 and considered as a *sensu stricto* microbiological research project by the Integrated Clinical Research Center of the Institut Pasteur (December 02, 2009). Clinical specimens were gathered and analyzed at the Institut Curie Hospital, Paris, France, a hospital dedicated to cancer diagnosis and treatment and data were analyzed anonymously.

#### Animal studies

Animals were housed in the Institut Pasteur animal facilities accredited by the French Ministry of Agriculture for performing experiments on live rodents (accreditation #A75-15 27, issued on November 12, 2004; and #A75-15 04, issued on May 22, 2008) in compliance with French and European regulations on care and protection of laboratory animals (EC Directive 86/609, French Law 2001-486, June 6, 2001). Protocols were approved by the veterinary staff of the Institut Pasteur animal facilities and were performed in compliance with NIH Animal Welfare Insurance #A5476-01 issued on July 02, 2007.

### Statistical analysis

Results for colony forming units and relative luminescence (ROI, p/S/cm^2^/sr) are mean +/− standard deviation. Box-and-whisker plot was used to display the degree of dispersion in data in case of *in vivo* studies and show the median (horizontal bar) and 25th/75th percentile. Statistical differences were evaluated using one-way ANOVA (Tukey multiple comparison test) included in Graphpad Prism Version 5.0c. Different treatment groups were considered statistically different if p values were lower than 0.05.

## Results

### Clinically relevant bioluminescent pathogens form in vitro biofilms in TIVAP

To develop a relevant model of biofilm infection, we first determined which bacterial pathogens most frequently contaminate human TIVAP in catheterized patients. For this, we performed microbiological evaluation of TIVAP removed for suspicion of infection in patients hospitalized at the Institut Curie Hospital, Paris, France [6], [21]. A total of 292 microorganisms were responsible for 279 TIVAP-associated infections. 66.4% of TIVAP were colonized by *Staphylococcus* spp. (31.1% by *S. aureus* and 18.5% by *S. epidermidis*), 16.4% by *Enterobacteriaceae* (including 5.8% by *E. coli*) and 12.3% by Pseudomonads (including 9.2% by *P. aeruginosa*) (Table S1). Based on these data, we obtained or constructed luminescent variants of clinical strains of *E. coli* (*E. coli* 55989 pAT881), *P. aeruginosa* (*P. aeruginosa* Lm1), *S. aureus* (*S. aureus* Xen36) and *S. epidermidis* (*S. epidermidis* Xen43) and showed that, except for weaker signals emitted by *S. epidermidis* Xen43, all other bacteria produced bioluminescent signals (ROI, p/S/cm^2^/sr) proportional to colony-forming units (CFU) (Figure S1).

We then evaluated the capacity of chosen luminescent bacteria to form *in vitro* biofilms in pediatric TIVAP using *in vitro* continuous flow system (Figure 1A). In these conditions, *E. coli*, *P. aeruginosa S. aureus* and *S. epidermidis* developed characteristic high-cell-density biofilm structures (Figure 1B), which correlated with high CFU counts and an increased luminescent signal (Figure 1CD).

These results therefore indicated that the four selected luminescent strains might be used to monitor bacterial colonization and biofilm formation in TIVAP.

### Non-invasive monitoring of long-term bacterial infection in rat-implanted TIVAP

To study medical device colonization in an *in vivo* context, we implanted TIVAP in rats (Figure S2). After 4 days of recovery, we checked for TIVAP sterility and functional connection with the rat venous system before investigating TIVAP bacterial colonization by *E. coli* 55989 pAT881, *P. aeruginosa* Lm1 *S. aureus* Xen36 and *S. epidermidis* Xen43 luminescent bacteria. We then injected 100 µL of bacterial inocula directly into the TIVAP port through externally decontaminated skin using a Huber needle. This inoculation volume was smaller than the TIVAP dead volume (250 µL), therefore avoiding injection into the bloodstream. We then monitored development and progression of TIVAP colonization as a function of luminescent signals.

**Figure 2:**
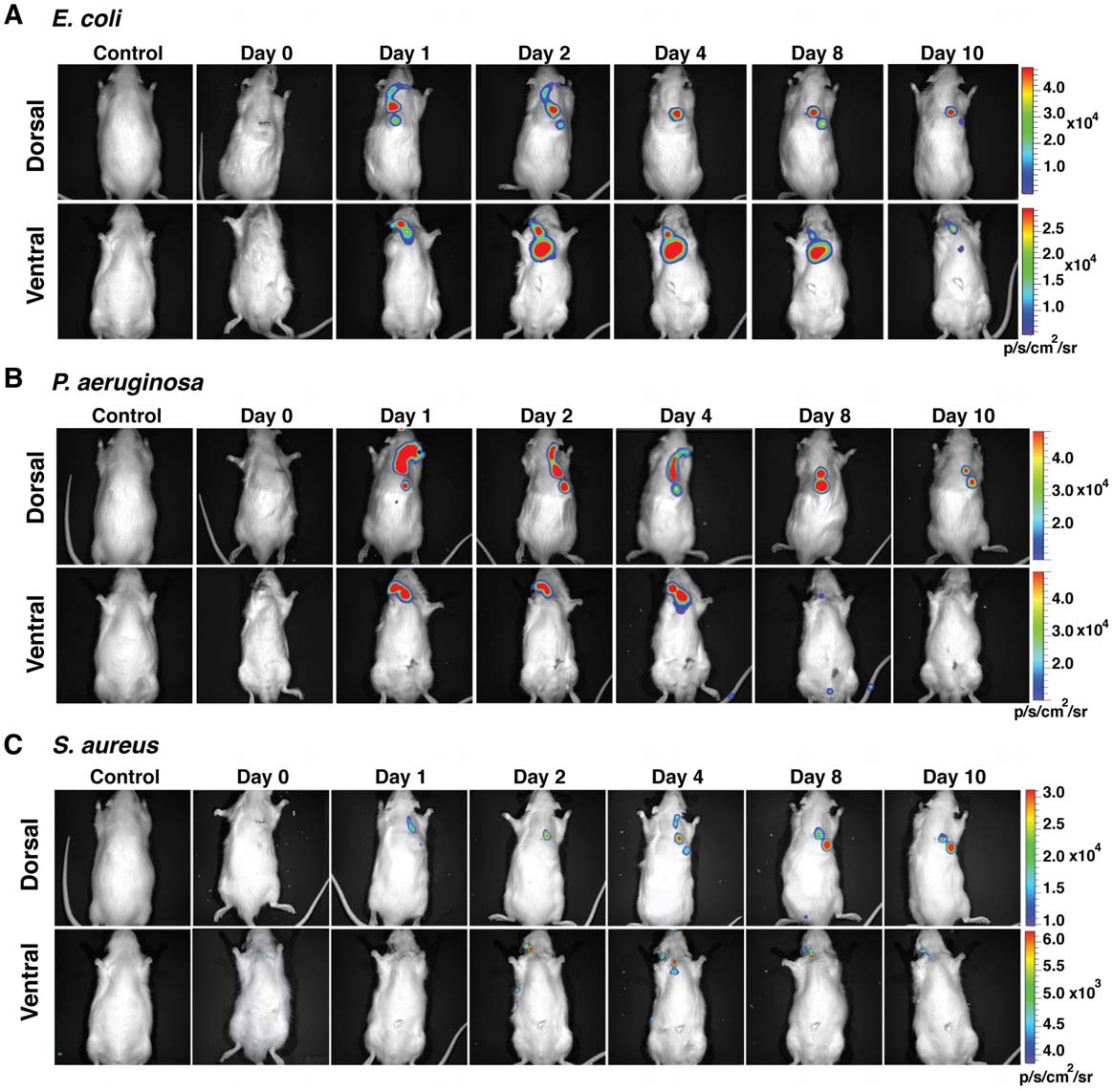
Monitoring of TIVAP colonization by *E. coli*, *P. aeruginosa* and *S. aureus*. The port was implanted subcutaneously at the dorsal midline and the catheter was inserted into the external jugular vein. Optimized doses of 10^4^ CFU (*E. coli*, n = 5) or 10^6^ CFU (*P. aeruginosa*, n = 7 and *S. aureus*, n = 6) in 100 µL were injected into the port and photon emission measured over a period of 10 days to monitor biofilm growth. Dorsal and ventral views of rats, showing progression of biofilm signals towards the catheter tip. Biofilm-associated infection was restricted to TIVAP by day 10. Control rat was catheterized but without bacterial inoculation. A representative animal is shown.

As described in Material and Methods section, we determined that injection of at least 10^4^ *E. coli* (n = 5), 10^6^ *P. aeruginosa* (n = 7) and 10^6^ *S. aureus* (n = 6) cells led to a clear luminescence signal, which progressed to the distal end of the catheter and peaked within 48–96 h before receding to the TIVAP (Figure 2A–C). In contrast, we could not identify a *S. epidermidis* (n = 5) injection titer leading to any detectable bioluminescence signals (data not shown). Finally, whereas most of our experiments were interrupted after 10 days post-inoculation (dpi), the bacterial luminescent signal in the port inoculated with luminescent *S. aureus* (n = 2), *E. coli* (n = 2) and *P. aeruginosa* (n = 2) could be detected up to a minimum of 60 dpi (Figure S3), thereby demonstrating establishment of long-term chronic colonization of TIVAP surgically implanted in rats.

### Bacterial colonization of rat-implanted TIVAP leads to formation of characteristic biofilms

**Figure 3:**
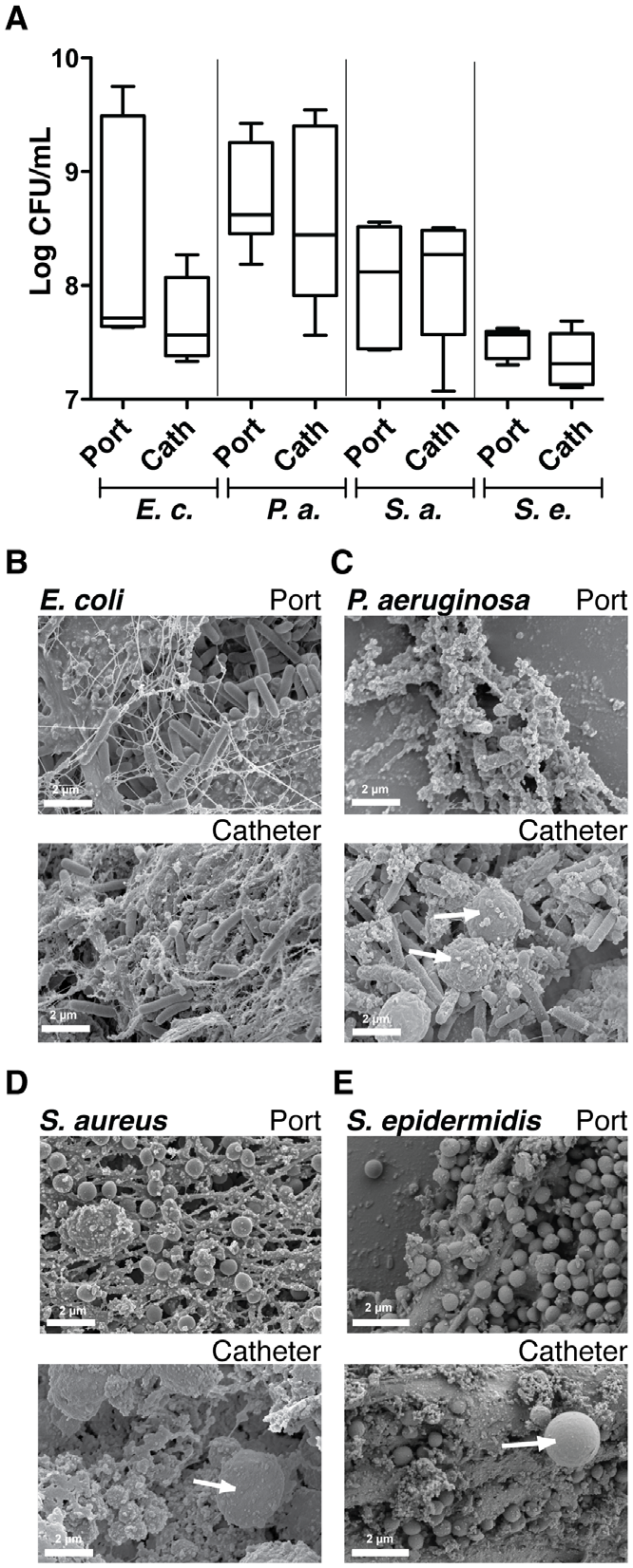
Bacterial colonization of TIVAP leads to biofilm formation. Rats were sacrificed 10 days post infection, TIVAP were removed aseptically and cells harvested from the catheter and port separately and plated on LB agar (E.c., *E. coli* n = 5 or P.a., *P. aeruginosa* n = 7) or TSB agar (S.a., *S. aureus* n = 6 or S.e., *S. epidermidis* n = 5) plates for CFU/mL (A). (B–E) SEM images confirming true biofilm formation in TIVAP *in vivo* in the port and catheter. Representative images are presented. White arrows indicate eukaryotic immune cells.

High bioluminescence signals were correlated to high bacterial titers within TIVAP. Counts of viable bacteria in the TIVAP after 10 dpi indicated ∼7.6 to 8.3-log CFU/mL (port) and ∼7.5 to 9.2-log CFU/mL (catheter) (Figure 3A) but a different co-relation between ROI and CFU/mL from port was obtained for each pathogen (Table S2). Moreover, electron microscopy (EM) showed that TIVAP colonized by *E. coli*, *P. aeruginosa* and *S. aureus* displayed characteristic bacterial biofilm structures (Figure 3B–D). In contrast with *in vitro* biofilms, bacteria were often embedded in a meshwork of host-derived fibrin-like material and associated with host cells potentially corresponding to host immune cells (see for example, Figure 3C–D–E). Interestingly, similar analyses performed on 10 dpi TIVAP inoculated with a high inoculum of 10^8^ *S. epidermidis* cells revealed clear biofilm formation (Figure 3E), but without an increase in CFU counts, suggesting that the absence of an *in vivo S. epidermidis* luminescent signal was not due to a lack of colonization ability by *S. epidermidis* Xen43 (Figure 3A).

### Clinical complications associated with use of TIVAP in rats

**Figure 4:**
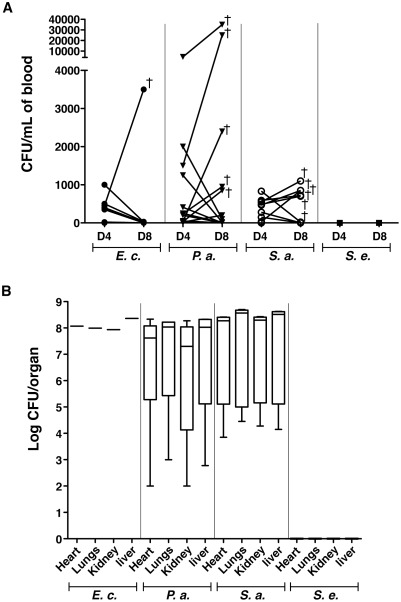
Clinically relevant complications associated with TIVAP biofilms. (A) Peripheral blood was harvested on day 4 (D4) and day 8 (D8) post-inoculation and plated on LB agar (E.c., *E. coli* n = 10 or P.a., *P. aeruginosa* n = 18) or TSB agar (S.a., *S. aureus* n = 14 or S.e., *S. epidermidis* n = 5) plates for CFU/mL. Bacteria were cleared by day 8 in most rats except for a few that suffered from systemic infection and died. Each dot represents one animal and cross (†) signifies the dead animal. (B) Organs were aseptically removed after sacrificing animals on day 10. Graph includes only rats that suffered from systemic infection and died. No bacteria were detected in the case of *S. epidermidis* (*S. e.*, n = 5), whereas 10% rats suffered from systemic infection and died due to *E. coli* (*E. c.*, n = 1/10) or ∼30% due to *P. aeruginosa* (*P. a.*, n = 6/18) and *S. aureus* (*S. a.*, n = 5/14) biofilms associated with TIVAP. Data are presented as box-and-whisker plots as previously described in material and methods.

To investigate whether TIVAP biofilms release bacteria, we monitored the presence of bacteria in the bloodstream in rats carrying colonized TIVAP. Peripheral blood was withdrawn from the retro-orbital sinuses of rats with inoculated TIVAP on days 4 and 8 post-inoculation and plated on either LB agar (for rats inoculated with *E. coli* and *P. aeruginosa*) or TSB agar plates (*S. aureus* and *S. epidermidis*). Although luminescence was restricted to the port and catheter after 48 h (cf. Figure 2), most rats, except for those colonized by *S. epidermidis*, showed varying numbers of CFU on day 4, thus indicating bacterial dissemination in bloodstream (Figure 4A). In several cases, implanted rats colonized with *S. aureus* (n = 5/14, ∼36%), *P. aeruginosa* (n = 6/18, ∼33%) and *E. coli* (n = 1/10, 10%) developed systemic infection leading to death between day 8 and 10, associated with massive organ bacterial colonization (Figure 4B).

At day 8, however, blood of all surviving TIVAP-infected rats was sterile; analysis of bioluminescence signals and CFU counts in lungs, heart, kidneys and liver aseptically removed from these rats was consistently negative, even after 60 dpi, with colonization restricted to the device (Figure 4A–B and S3). These results indicated that our model mimicked chronic colonization of implanted devices, with occasional catheter-related systemic bloodstream infections.

In clinical situations, repeated intradermal needle puncture during access to TIVAP is also associated with subcutaneous bacterial infection around the port area [25]. Consistently, we found that 20% of TIVAP-implanted rats inoculated with *P. aeruginosa* or *S. aureus* (but not with *E. coli*) developed subcutaneous port pocket infection. In addition to iatrogenic TIVAP-related infections, bacteria from the patient bloodstream also constitute a source of central venous catheter colonization. To test whether TIVAP implanted in rats can also be colonized via a hematogenous route of infection, we injected 5×10^8^ luminescent *S. aureus* bacteria through the lateral tail vein of TIVAP-implanted rats. Although no signs of systemic infection were observed in rats 3 days post-injection, 30% (n = 2/6) displayed a bioluminescent signal and bacterial colonization at the catheter tip of the TIVAP (Figure S4).

These results demonstrated that TIVAP implanted rats experienced clinical situations such as biofilm-related bloodstream infections, organs metastasis and port-pocket infections.

### Role of the rat immune system in control of TIVAP biofilm infections

Confinement of high cell density biofilms to the TIVAP and clearance of bloodstream bacteria several days after inoculation suggested active *in vivo* control of device-related infections. To test the role of the rat immune system in this control, we transiently immunosuppressed implanted rats prior to inoculation using cyclophosphamide, a commonly used anti-cancer drug that causes neutropenia and lowers the number of other white blood cells [26]. Consistently, implanted cyclophosphamide-treated rats displayed a 70% decrease in total blood leukocytes.

**Figure 5:**
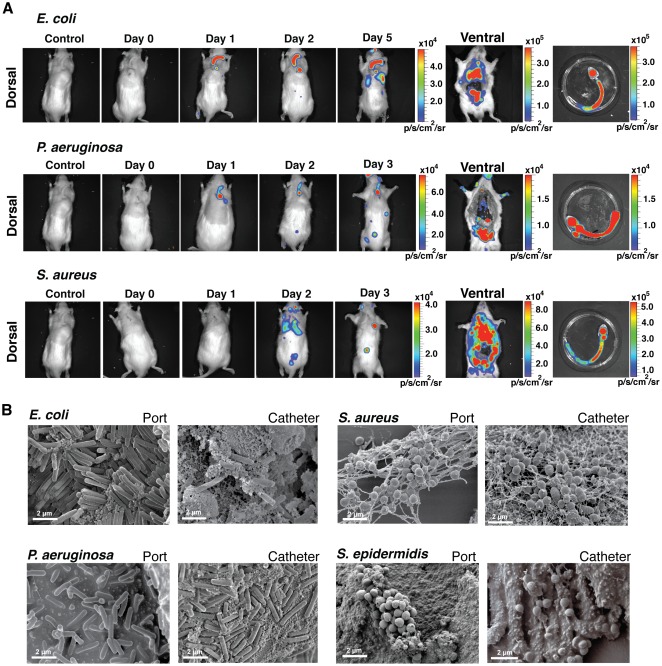
Immunosuppression led to fatal biofilm infection. (A) 10^2^ CFU in 100 µL of the different bacteria were injected into the port of TIVAP implanted in cyclophosphamide-treated rats (100 mg/kg day -4 and 50 mg/kg day -1 of inoculation) and photon emission was monitored up to the death of the animals on days 3/5 to evaluate biofilm formation and associated infection. TIVAP were aseptically removed from dead animals and photon emission measured for both the animal and the extracted TIVAP. Control rat was a catheterized and cyclophosphamide-treated but without bacterial inoculation. (B) SEM images of TIVAP (port and catheter) infected with 4 clinical strains used in this study. Number of rats (n) used in the experiment, n = 4 for each strain.

We observed that inoculation of TIVAP in immunosuppressed rats with as low as 10^2^ cells (in contrast to 10^4^ to 10^8^ in immunocompetent rats) led to a strong luminescent signal in inoculated TIVAP at day 1 post-inoculation, except for *S. epidermidis*, which could not be monitored by luminescence (Figure 5A). Strikingly, viable bacteria counts and electron microscopy analyses revealed strong biofilm development for all four bacteria tested in TIVAP aseptically removed from immunosuppressed rats, with approximately ∼9-log CFU/mL recovered from ports or catheters (Figure S5). EM analyses also showed that substantially fewer immune cells could be found in *E. coli*, *P. aeruginosa*, *S. aureus* and *S. epidermidis* biofilms formed in TIVAP ports and catheters of immunosuppressed rats compared to biofilm formed in TIVAP implanted in immunocompetent rats (Figure 5B).

In addition, whereas control (non-infected) immunosuppressed rats survived, all inoculated rats ultimately died of massive acute systemic infection between days 3 and 5, with bioluminescent signals progressing from the TIVAP throughout the body for *E. coli*, *P. aeruginosa* and *S. aureus* (Figure 5A). Collection of peripheral blood on the day of the animals' death showed that cyclophosphamide-treated rats were indeed bacteremic (Figure S5). Metastatic infectious disease was confirmed by detection of strong bioluminescence signals and a high CFU count in aseptically removed organs (lungs, heart, kidneys, liver) (Figure S5).

These results showed that immunosuppressed rats were unable to restrict the extent and outcome of TIVAP-related infections. Interestingly, ∼30% of immunocompetent rats that spontaneously developed fatal systemic *P. aeruginosa* and *S. aureus* infection (see above and Figure 4) showed an exacerbated inflammatory response, as indicated by a significant increase in total white blood cells and granulocytes in peripheral blood compared to immunocompetent rats that controlled their TIVAP infection (Figure S6).

Taken together, these results demonstrated that our model enabled studying the role of the rat immune system in development of acute biofilm-mediated systemic infection caused by bacterial pathogens.

### Evaluation of the anti-biofilm antibiotic lock in TIVAP

**Figure 6:**
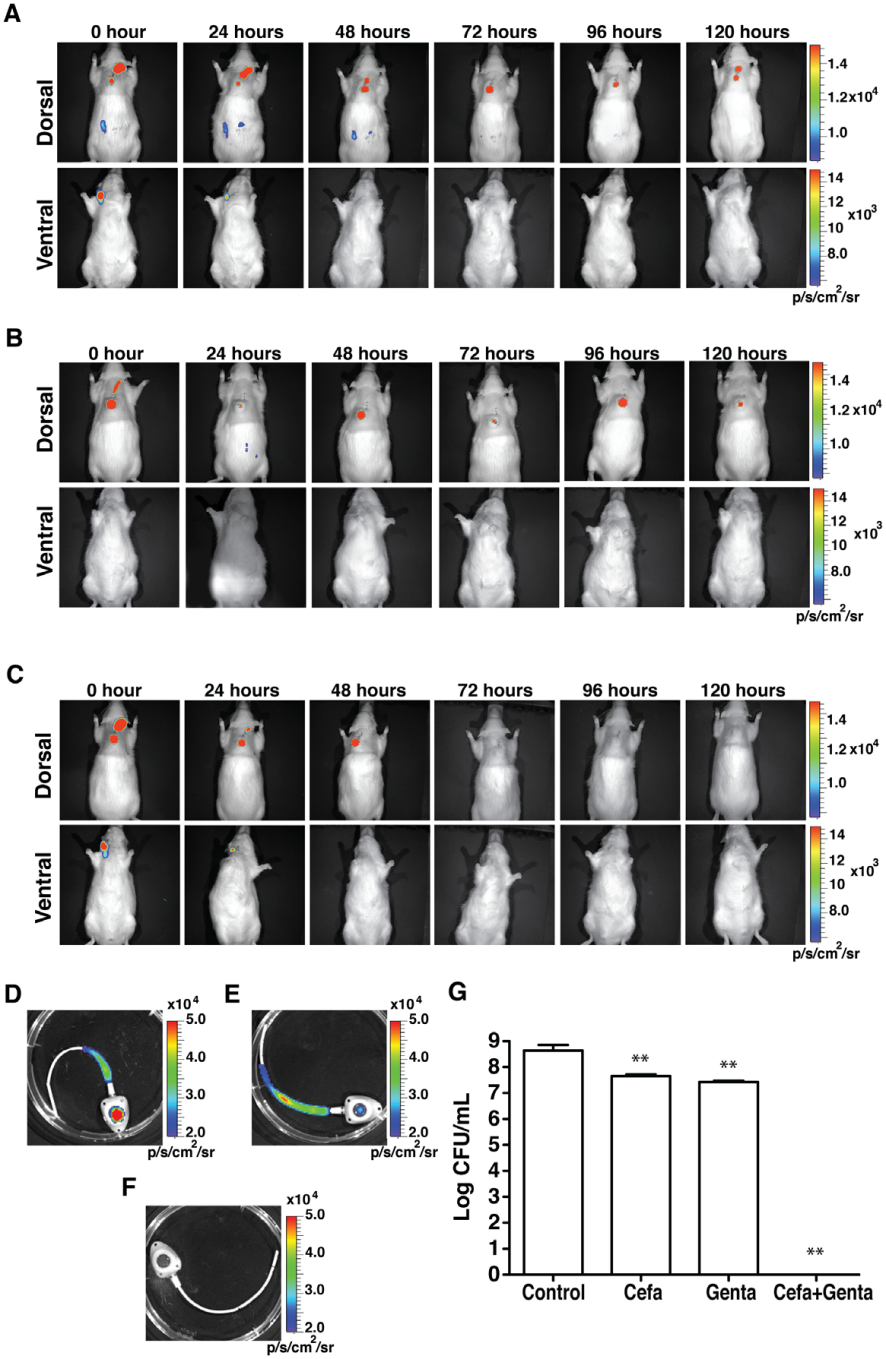
*In vivo* efficacy of ALT. ALT was instilled in the implanted colonized TIVAP (0 h) and associated with systemic vancomycin to treat *S. aureus* biofilm colonization. ALT was renewed every 24 h and its efficacy was monitored as photon emissions. Rats (n = 4, for each treatment) were sacrificed after 120 h of treatment and analyzed. (A) 5 mg/mL cefazolin ALT. (B) 1 mg/mL gentamicin ALT. (C) Combined cefazolin and gentamicin ALT (1∶1 v/v). (D–E) TIVAP were aseptically removed and photon emission due to remnant biofilm measured. (D) Cefazolin-treated TIVAP, (E) gentamicin-treated TIVAP and (F) TIVAP treated with cefazolin and gentamicin combination. In (A) to (F) representative experiments are shown. (G) TIVAP was extracted after treatment and cells were harvested and plated for CFU/mL. All the values are mean +/− standard deviation. Statistical analysis was done using one-way analysis of variance (ANOVA) using Graphpad Prism version 5.0c. p value<0.05 considered significant, *** (p<0.0001), ** (p<0.001) and * (p<0.05).

To evaluate the clinical relevance of our *in vivo* model, we chose a widely used anti-biofilm method called the antibiotic lock therapy (ALT) using cefazolin and gentamicin in TIVAP inoculated with *S. aureus* Xen36. ALT corresponds to instillation of a high concentration of antibiotics into the lumen of the catheter for a dwelling time varying between 12 and 24 h [27]. In clinical situations, ALT is associated with systemic antibiotic treatment to prevent bloodstream infection originating from the TIVAP. We observed that, while systemic vancomycin treatment avoided bloodstream infections, ALT solutions composed of cefazolin or gentamicin alone, mixed with heparin and used for 5 consecutive days with daily replacement, did not completely eradicate *S. aureus* biofilms in TIVAP, as indicated by the persistence of a luminescence signal and the presence of 10^7^–10^8^ CFU/ml in removed TIVAP (Figure 6). In contrast, use of the antibiotic lock solution consisting of a combination of cefazolin and gentamicin (1∶1 V/V in heparin 2500 IU/mL) very effectively eradicated TIVAP-associated biofilm (Figure 6). Interestingly, when ALT was applied without systemic treatment, approximately 30% of immunocompetent rats were unable to clear bloodstream infection originating from TIVAP-associated biofilm bacteria flushed during the ALT procedure, and rapidly died after instilling ALT, therefore pointing to the risk of use of ALT treatment without an associated systemic antibiotic treatment (Figure S7).

These results demonstrated that our *in vivo* model of biofilm infection constitutes a relevant tool for evaluating the efficiency of ALT protocols and investigating other clinical issues associated with use of central venous catheters.

## Discussion

We developed a novel *in vivo* model of bacterial colonization in TIVAP and demonstrated that it constitutes a clinically relevant tool for studying both microbial and host contribution to catheter-related biofilm infections. TIVAP, developed in 1982 to replace external catheters for antineoplastic chemotherapy, are commonly used medical devices that are surgically implanted at chest level; they are routinely employed to facilitate long-term repetitive intravenous therapy [28]. Despite significant progress in clinical handling of these catheters, biofilm development resulting from external (skin and environment) or endogenous (bloodstream infection) contamination remains a poorly understood process with severe clinical ramifications [6], [29]. The pediatric-sized TIVAP used in our study are well adapted to small laboratory mammals; their subcutaneous implantation prevents rapid accidental removal by implanted animals, which obviates the need for cumbersome restrainer jackets and enables long-term studies [18], [30], [31]. Unlike other venous catheters, TIVAP are closed devices, thereby considerably reducing the risk of unplanned contamination and making them particularly amenable to controlled reproducible long-term animal infection studies. We followed bacterial colonization using bioluminescence, a well-established technology for real-time and *in vivo* bacterial monitoring in animal models [32]. Here we show that, in addition to CFU count and electron microscopy, bioluminescence can also accurately reflect the level of bacterial colonization of the port, catheter and organs. Both the literature and our own microbiological analysis of infected clinical TIVAP showed that staphylococci account for more than 60% of all identified pathogens in central venous-catheter-related infections [33]. While luminescent *S. aureus* could be used for quantitative *in vitro* and *in vivo* monitoring of bacterial colonization in TIVAP, the level of signals emitted by the only currently available bioluminescent strain of *S. epidermidis* was insufficient to permit its *in vivo* monitoring in TIVAP. Nevertheless, since *S. epidermidis* is one of the leading causes of central-venous-catheter-associated biofilm infections in a hospital environment, especially in critically ill patients [34], we also used non-luminescent-based approaches including microscopy and CFU count; we showed that *S. epidermidis* is an efficient TIVAP colonizer both *in vitro* and *in vivo*. We also demonstrated the capacity of *E. coli* and *P. aeruginosa* pathogenic strains to colonize TIVAP. These Gram-negative bacteria are often overlooked in studies using catheter-related infection models, but recent data raise concern about their increased importance in the microbiological epidemiology of oncology patients, in addition to multiresistance associated with those pathogens [35], [36], [37], [38], [39].

We demonstrated that *in vitro* and *in vivo* colonization of TIVAP by the four tested bacteria led to formation of biofilms displaying characteristic high cell density as shown by scanning electron microscopy of both TIVAP septa and catheters, along with CFU count. In clinical settings, the formation of pathogenic biofilms in central venous catheters is responsible for bloodstream infections. We reproducibly observed that controlled inoculation of implanted TIVAP enabled biofilm development that occasionally led to lethal systemic infections. In most cases, however, bacteria released from persistent TIVAP biofilms were rapidly cleared from the peripheral blood and organs of immunocompetent rats. This situation could be maintained for up to 120 days, therefore reproducing the presence of chronic biofilms in medical devices, which are considered important potential infection reservoirs [25].

The persistence of biofilms in these devices for such a long period of time clearly pointed to the incapacity of the host immune system to clear such structured bacterial communities despite the presence of immune cells in the observed biofilms. However, colonization of TIVAP implanted in immunosuppressed rats by *E. coli*, *P. aeruginosa*, *S. aureus* or *S. epidermidis* bacteria consistently led to rapid acute systemic infection and death, therefore illustrating the importance of host immune response in controlling bacterial metastasis originating from persistent biofilms in colonized TIVAP. Even in the case of poorly luminescent *S. epidermidis*, in accordance with a previous study using subcutaneously implanted catheter segments [14], we showed that *S. epidermidis* biofilm-associated infection was very efficiently cleared by the rat immune system [34]. Our results are therefore consistent with clinical and experimental evidence in support of a key contribution of host immune system factors to the outcome of catheter-related infections [40].

Besides mimicking of chronic and acute biofilm infections, rats implanted with pediatric TIVAP also developed common clinical complications such as port pocket infection and catheter tip colonization via a hematogenous route.

Development of strategies to prevent or treat indwelling medical device biofilm infections relies heavily on the availability of relevant *in vivo* models amenable to preclinical trial evaluation [6], [10], [11], [38], [41], [42]. Our results demonstrated that our model of TIVAP-related biofilm infections might be a valuable tool for assessing therapeutic strategies against device-related biofilm infection. In this study, we evaluated the efficiency of the antibiotic lock therapy (ALT), a widely used strategy recommended in case of uncomplicated catheter-related bloodstream infection (CRBSI) caused by coagulase-negative staphylococci and Gram-negative bacteria [43], [44], [45]. In the case of *S. aureus* CRBSI, treatment failure and hematogenous complications are frequent [44], [46]. We therefore focused on this difficult-to-treat pathogen and showed that clinically proposed high concentrations of gentamicin or cefazolin alone did not eradicate *S. aureus* biofilm, as indicated by the persistence of luminescent signals and high CFU counts in TIVAP after treatment. However, combined antibiotics acted synergistically and led to clearance of the dense biofilms formed in TIVAP within 72 h of treatment. While this clearly illustrates the high tolerance of biofilms towards antibiotics, it also suggested that our model could be used to assess the curative or preventive efficiency of innovative combinatorial ALT solutions or antibacterial/anti-adhesive impregnated catheters. In addition to new antibiofilm approaches, our model permits evaluation of other aspects of clinical handling of TIVAP, including recolonization after replacement of contaminated TIVAP, flush protocols and antithrombotic monitoring.

In conclusion, we describe a new *in vivo* model that would contribute to a better understanding of device-related infections by addressing numerous clinically relevant issues, including the impact of the immune system on the kinetic of establishment and control of biofilm-related infections. This model will also allow assessment of diagnostic or therapeutic anti-biofilm and thrombosis approaches, as well as optimization of long-term management of implanted medical devices.

## Supporting Information

Figure S1 **Increase in bacterial count is correlated with increased luminescence and CFU.** Bacteria were grown in liquid medium (LB for *E. coli* and *P. aeruginosa* and TSB glucose for *S. aureus* and *S. epidermidis*) and samples were harvested over time to evaluate bacterial concentration (–•–, CFU/mL) and relative bioluminescence (–□–, ROI, p/S/cm^2^/sr). (A–C) For *E. coli*, *P. aeruginosa* and *S. aureus* bioluminescence followed growth and remained relatively high. (D) *S. epidermidis* showed a marked decrease in bioluminescence over time with increasing CFU and displayed lower levels of bioluminescence than the other bacteria. All the values are mean +/− standard deviation of 3 values.
(TIF)

Figure S2 **Surgical implantation of TIVAP in rats. TIVAP were surgically implanted in CD/SD (IGS: Crl) rats.** (A) Surgery was performed under laminar air flow and aseptic conditions were maintained throughout the surgical procedure. (B) Rats were briefly kept in an isoflurane chamber to calm down and injected intraperitoneally with a ketamine/xylazine/acepromazine mixture to complete sedation and analgesia before starting the procedure. (C) After shaving and skin disinfection, an incision was made at the dorsal midline. (D) The catheter from the pediatric TIVAP was cut at a 10/11 cm length. (E). A subcutaneous pocket was created and the port carefully inserted before being held intact by sutures (F). (G) An incision was made in the neck area on the ventral side and a Huber needle connected to a tuberculin syringe was inserted into the port. (H) The catheter was tunneled under the skin with the help of a tunneling rod provided with the TIVAP kit. (I) The external jugular vein was exposed and two cotton threads inserted beneath the vein on the proximal and distal sides. The catheter was cut at a slant. (J) A small incision was made in the jugular vein and the catheter inserted into the vein and pushed up to the superior vena cava. (K) Blood reflux was checked by pulling the blood carefully and the TIVAP was flushed with 1X PBS. The catheter was held in place by tying the threads. Suturing of both the dorsal and ventral sides closed the wounds (L) and (M). Lidocaine and betadine were applied to the wounds and rats were allowed to recover for 4 days prior to inoculation. Surgical wounds healed within a week (N) and (O).
(TIF)

Figure S3 **Long-term biofilms formed in TIVAP implanted in rats.** Several rats (n = 3, for each strain) with implanted TIVAP were monitored for persistent infection over a long period of time. Persistent biofilm growth was observed for *E. coli* up to 60 days (A), for *P. aeruginosa* up to 65 days (B) and for *S. aureus* up to a period of 128 days (C). (D) Rats were sacrificed and TIVAP removed to confirm the presence of biofilms by luminescence.
(TIF)

Figure S4 **Mimicking of hematogenous colonization.** Central venous catheters were colonized by bacteria from other infection routes, including bloodstream infection. (A) 5×10^8^ CFU/500 µL 1X PBS were injected through the tail vein of TIVAP-implanted rats (n = 6). Photon emission due to bacterial colonization on the TIVAP was monitored and appeared to localize at the catheter tip. (B) Localization of bacterial biofilm on the tip of the TIVAP (n = 2/6) was confirmed by removal on day 3 post intravenous injection and imaging of the catheter. Approximately 10^4^ CFU were detected after resuspension of 1 cm of the catheter tip. Representative images are presented.
(TIF)

Figure S5 **Immunosuppression led to fatal biofilm borne infection due to bacteremia and severe organ infection.** (A) TIVAP was surgically removed and cells harvested from the catheter (Cath) and port separately and plated for CFU/mL. Data are presented as box-and-whisker plots as previously described in Material and Methods. (B) Peripheral blood was withdrawn by puncturing the retro-orbital sinus on the final day of the experiment and plated for CFU/mL. (C) Organs were aseptically removed on the last day of the experiment, homogenized and plated for CFU/mL. All organs were heavily contaminated (all four pathogenic bacteria) with up to 10^9^ CFU/mL. CFU were estimated on LB agar (E.c., *E. coli*) or P.a., *P. aeruginosa*) or TSB agar (S.a., *S. aureus* or S.e., *S. epidermidis*) plates. Data are presented as box-and-whisker plots as previously described. Number of rats (n) used in the experiment, n = 4 for each strain.
(TIF)

Figure S6 **Exacerbation of immune cells in peripheral blood in systemically infected rats.** Blood was collected from the rats by retro-orbital puncture as described in material and methods and analysed using ABC vet automated blood analysis machine. (A) Total leukocyte count for control (uninfected rats, n = 6), *E. coli* (n = 3), *P. aeruginosa* (healthy, n = 5; sick, n = 4) and *S. aureus* (healthy, n = 3 and sick n = 3). (B) Granulocyte count in peripheral blood for control (uninfected rats, n = 6), *E. coli* (n = 3), *P. aeruginosa* (healthy, n = 5; sick, n = 4) and *S. aureus* (healthy, n = 3 and sick n = 3). Statistical analysis was done using one-way analysis of variance (ANOVA) using Graphpad Prism version 5.0c. Except when indicated by connectors statistical comparisons were made with control. p value<0.05 considered significant, *** (p<0.0001), ** (p<0.001) and * (p<0.05).
(TIF)

Figure S7 **ALT treatment of** ***S. aureus*** **biofilms can lead to systemic infection in animals.** Treatment of biofilm colonization in TIVAP-implanted rats with ALT led to systemic infection when not combined with systemic treatment. (A) ALT (0 h) was instilled on day 4 post-biofilm colonization in TIVAP. ALT treatment led to flushing of bacteria into the bloodstream for 30% of treated rats and led to organ infection (n = 3/9). (B) Rat showing systemic infection after 168 h of ALT treatment leading to its death. TIVAP was removed and luminescence measured, confirming persistent biofilm colonization. A representative experiment is shown.
(TIF)

Table S1 **Microbiological analysis of TIVAP removed from human patients (n = 279) with suspected catheter-related infection.**
(DOCX)

Table S2 ***In vivo*** **relation between ROI (p/s/cm^2^/sr) and CFU/ml in port of implanted TIVAP at 10 dpi.**
(DOCX)
