# Supplementary figures and images for: A Rat Model of Central Venous Catheter to Study Establishment of Long-Term Bacterial Biofilm and Related Acute and Chronic Infections
Source: PLoS One. 2012 May 16;7(5):e37281. doi: 10.1371/journal.pone.0037281 (PMC3353920; doi:10.1371/journal.pone.0037281)

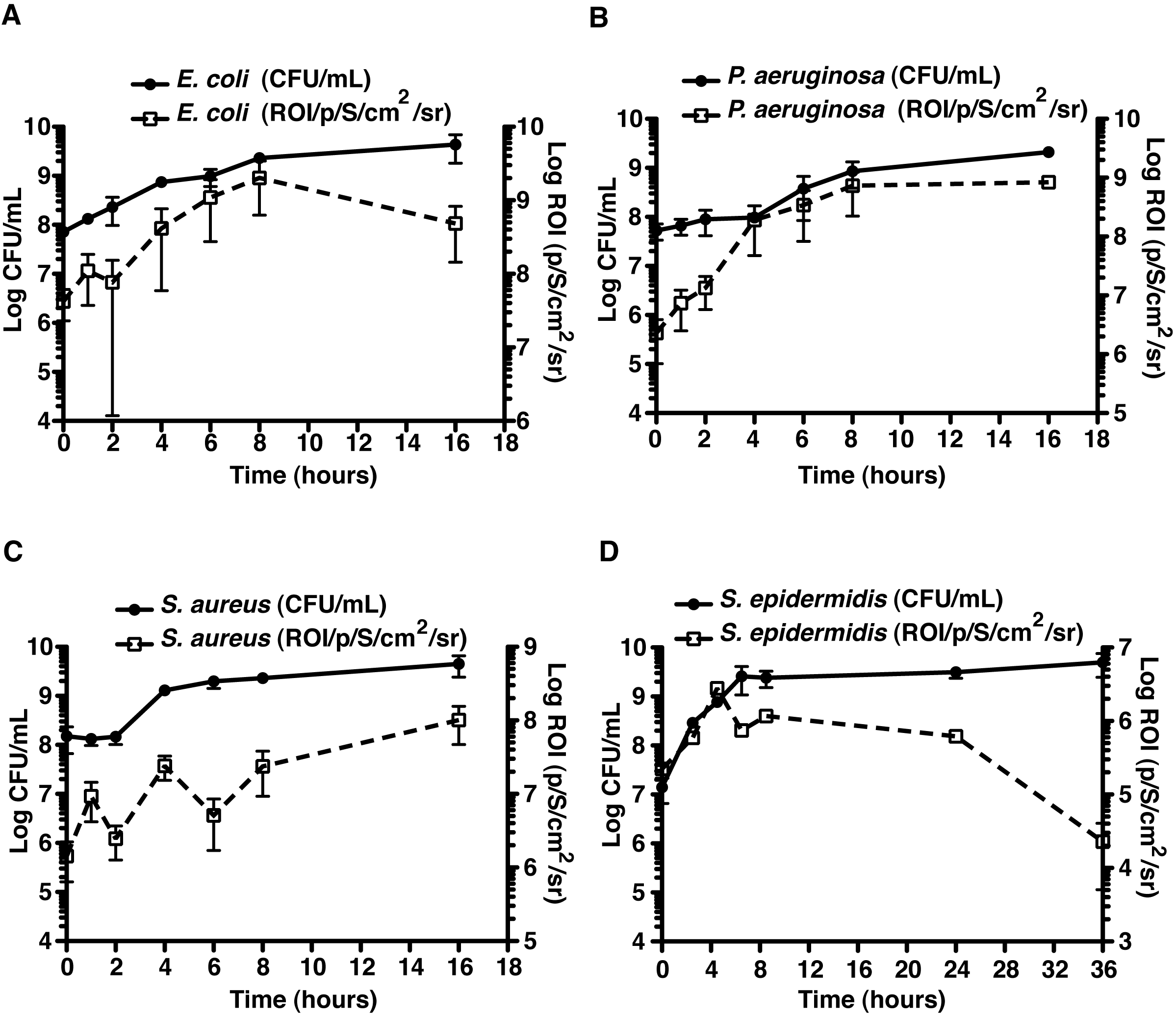

Supplement: Figure S1 — Increase in bacterial count is correlated with increased luminescence and CFU. Bacteria were grown in liquid medium (LB for E. coli and P. aeruginosa and TSB glucose for S. aureus and S. epidermidis) and samples were harvested over time to evaluate bacterial concentration (–•–, CFU/mL) and relative bioluminescence (–□–, ROI, p/S/cm2/sr). (A–C) For E. coli, P. aeruginosa and S. aureus bioluminescence followed growth and remained relatively high. (D) S. epidermidis showed a marked decrease in bioluminescence over time with increasing CFU and displayed lower levels of bioluminescence than the other bacteria. All the values are mean +/− standard deviation of 3 values. (TIF) [file pone.0037281.s001.tif]

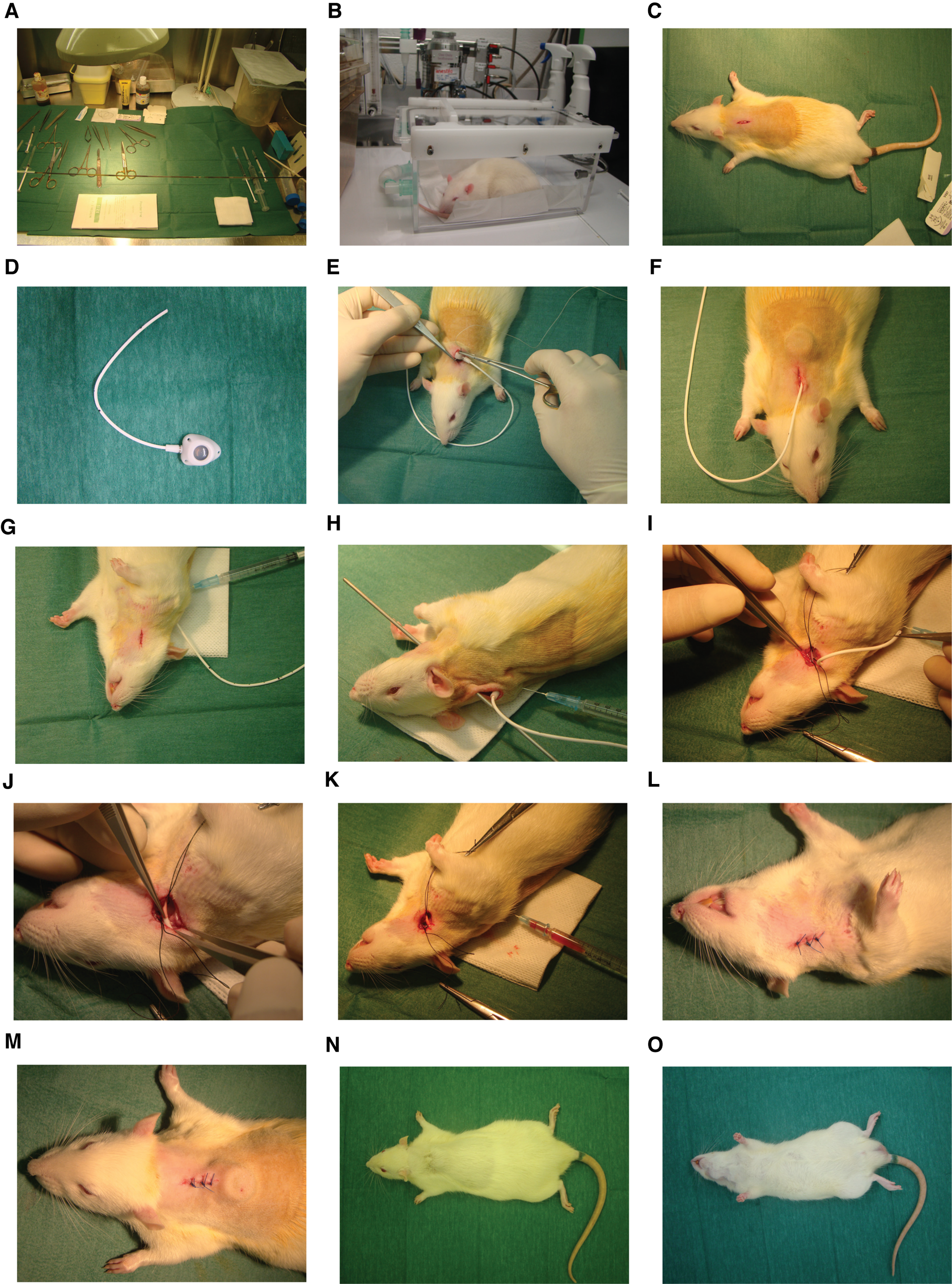

Supplement: Figure S2 — Surgical implantation of TIVAP in rats. TIVAP were surgically implanted in CD/SD (IGS: Crl) rats. (A) Surgery was performed under laminar air flow and aseptic conditions were maintained throughout the surgical procedure. (B) Rats were briefly kept in an isoflurane chamber to calm down and injected intraperitoneally with a ketamine/xylazine/acepromazine mixture to complete sedation and analgesia before starting the procedure. (C) After shaving and skin disinfection, an incision was made at the dorsal midline. (D) The catheter from the pediatric TIVAP was cut at a 10/11 cm length. (E). A subcutaneous pocket was created and the port carefully inserted before being held intact by sutures (F). (G) An incision was made in the neck area on the ventral side and a Huber needle connected to a tuberculin syringe was inserted into the port. (H) The catheter was tunneled under the skin with the help of a tunneling rod provided with the TIVAP kit. (I) The external jugular vein was exposed and two cotton threads inserted beneath the vein on the proximal and distal sides. The catheter was cut at a slant. (J) A small incision was made in the jugular vein and the catheter inserted into the vein and pushed up to the superior vena cava. (K) Blood reflux was checked by pulling the blood carefully and the TIVAP was flushed with 1X PBS. The catheter was held in place by tying the threads. Suturing of both the dorsal and ventral sides closed the wounds (L) and (M). Lidocaine and betadine were applied to the wounds and rats were allowed to recover for 4 days prior to inoculation. Surgical wounds healed within a week (N) and (O). (TIF) [file pone.0037281.s002.tif]

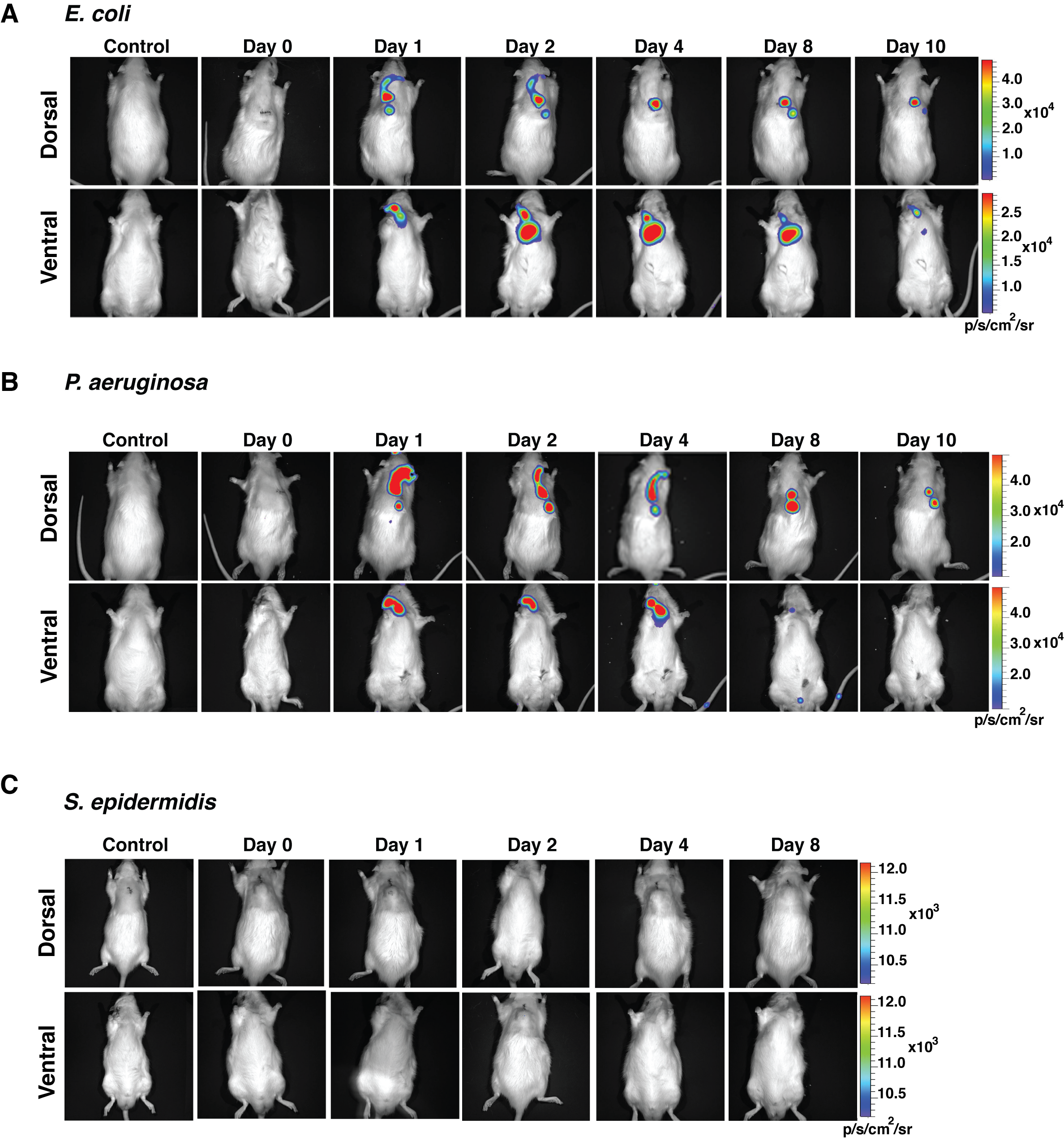

Supplement: Figure S3 — Long-term biofilms formed in TIVAP implanted in rats. Several rats (n = 3, for each strain) with implanted TIVAP were monitored for persistent infection over a long period of time. Persistent biofilm growth was observed for E. coli up to 60 days (A), for P. aeruginosa up to 65 days (B) and for S. aureus up to a period of 128 days (C). (D) Rats were sacrificed and TIVAP removed to confirm the presence of biofilms by luminescence. (TIF) [file pone.0037281.s003.tif]

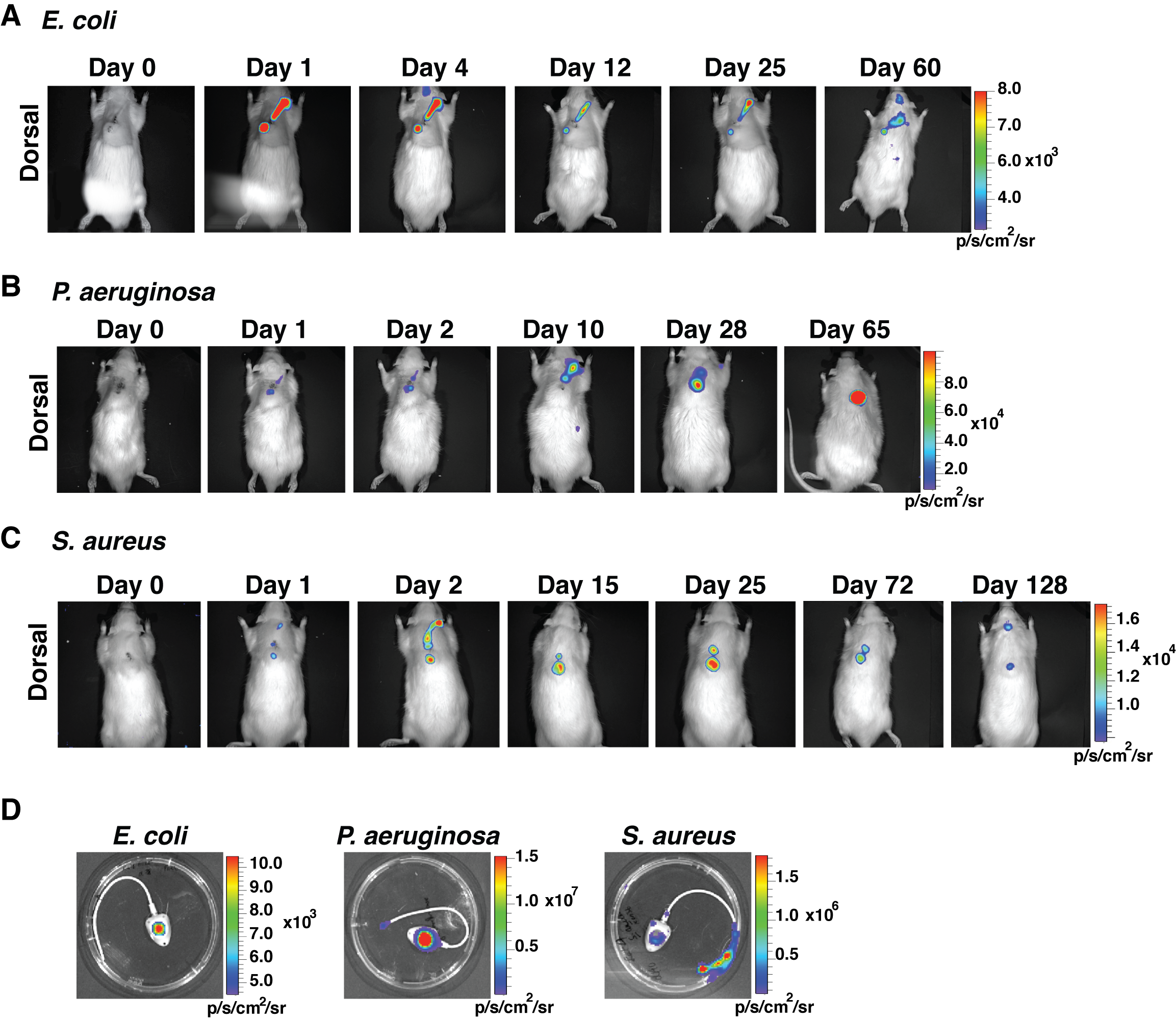

Supplement: Figure S4 — Mimicking of hematogenous colonization. Central venous catheters were colonized by bacteria from other infection routes, including bloodstream infection. (A) 5×10^8^ CFU/500 µL 1X PBS were injected through the tail vein of TIVAP-implanted rats (n = 6). Photon emission due to bacterial colonization on the TIVAP was monitored and appeared to localize at the catheter tip. (B) Localization of bacterial biofilm on the tip of the TIVAP (n = 2/6) was confirmed by removal on day 3 post intravenous injection and imaging of the catheter. Approximately 104 CFU were detected after resuspension of 1 cm of the catheter tip. Representative images are presented. (TIF) [file pone.0037281.s004.tif]

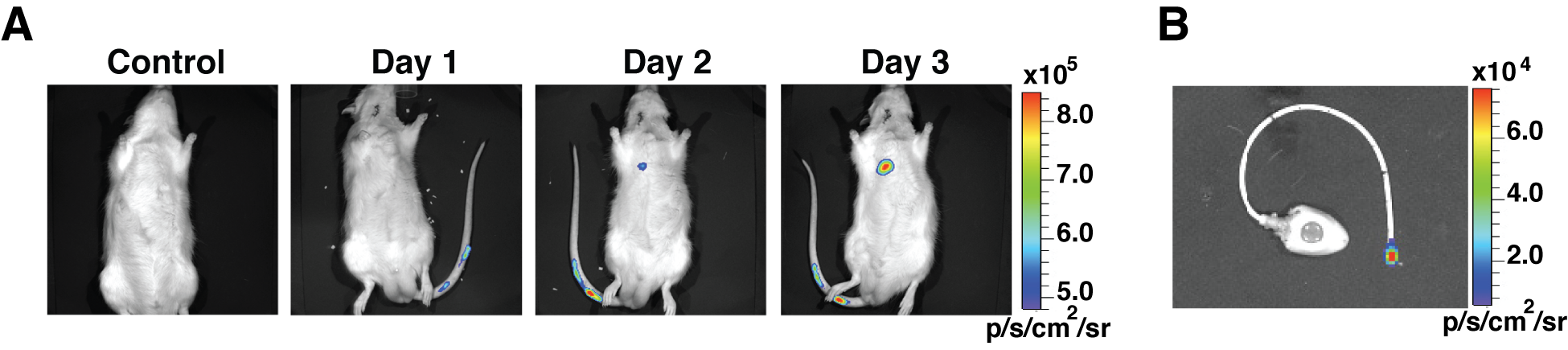

Supplement: Figure S5 — Immunosuppression led to fatal biofilm borne infection due to bacteremia and severe organ infection. (A) TIVAP was surgically removed and cells harvested from the catheter (Cath) and port separately and plated for CFU/mL. Data are presented as box-and-whisker plots as previously described in Material and Methods. (B) Peripheral blood was withdrawn by puncturing the retro-orbital sinus on the final day of the experiment and plated for CFU/mL. (C) Organs were aseptically removed on the last day of the experiment, homogenized and plated for CFU/mL. All organs were heavily contaminated (all four pathogenic bacteria) with up to 109 CFU/mL. CFU were estimated on LB agar (E.c., E. coli) or P.a., P. aeruginosa) or TSB agar (S.a., S. aureus or S.e., S. epidermidis) plates. Data are presented as box-and-whisker plots as previously described. Number of rats (n) used in the experiment, n = 4 for each strain. (TIF) [file pone.0037281.s005.tif]

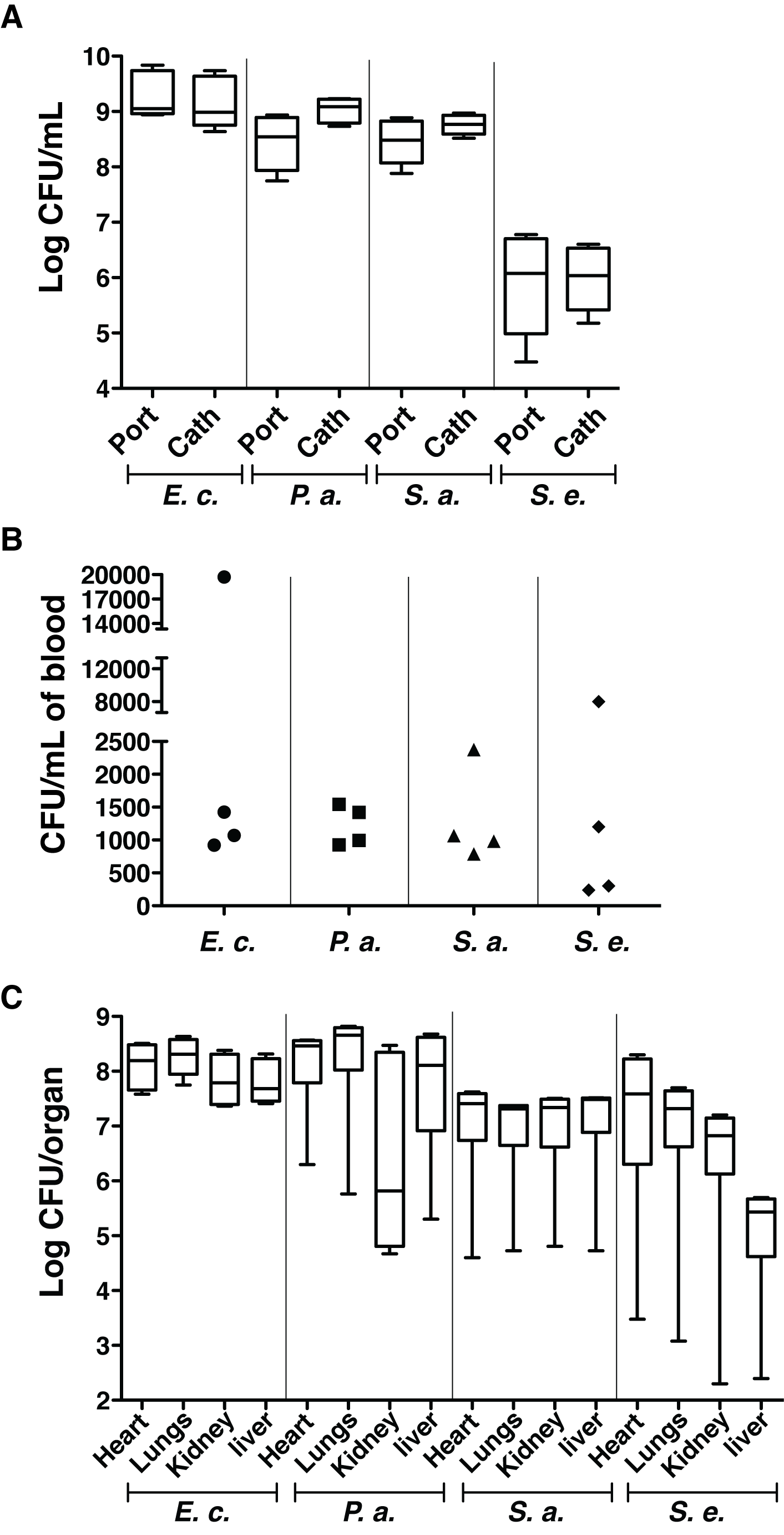

Supplement: Figure S6 — Exacerbation of immune cells in peripheral blood in systemically infected rats. Blood was collected from the rats by retro-orbital puncture as described in material and methods and analysed using ABC vet automated blood analysis machine. (A) Total leukocyte count for control (uninfected rats, n = 6), E. coli (n = 3), P. aeruginosa (healthy, n = 5; sick, n = 4) and S. aureus (healthy, n = 3 and sick n = 3). (B) Granulocyte count in peripheral blood for control (uninfected rats, n = 6), E. coli (n = 3), P. aeruginosa (healthy, n = 5; sick, n = 4) and S. aureus (healthy, n = 3 and sick n = 3). Statistical analysis was done using one-way analysis of variance (ANOVA) using Graphpad Prism version 5.0c. Except when indicated by connectors statistical comparisons were made with control. p value<0.05 considered significant, *** (p<0.0001), ** (p<0.001) and * (p<0.05). (TIF) [file pone.0037281.s006.tif]

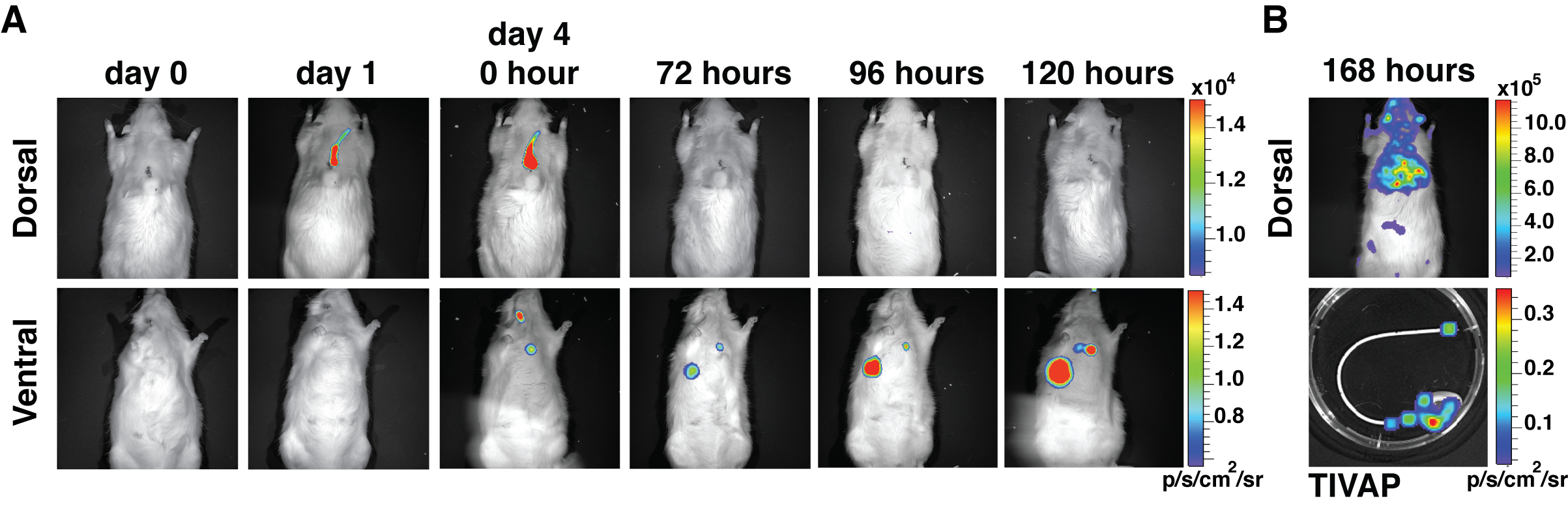

Supplement: Figure S7 — ALT treatment of S. aureus biofilms can lead to systemic infection in animals. Treatment of biofilm colonization in TIVAP-implanted rats with ALT led to systemic infection when not combined with systemic treatment. (A) ALT (0 h) was instilled on day 4 post-biofilm colonization in TIVAP. ALT treatment led to flushing of bacteria into the bloodstream for 30% of treated rats and led to organ infection (n = 3/9). (B) Rat showing systemic infection after 168 h of ALT treatment leading to its death. TIVAP was removed and luminescence measured, confirming persistent biofilm colonization. A representative experiment is shown. (TIF) [file pone.0037281.s007.tif]
