# Supplementary material for: A Rat Model of Central Venous Catheter to Study Establishment of Long-Term Bacterial Biofilm and Related Acute and Chronic Infections
Source: PLoS One. 2012 May 16;7(5):e37281. doi: 10.1371/journal.pone.0037281 (PMC3353920; doi:10.1371/journal.pone.0037281)
Supplement: Table S1 — Microbiological analysis of TIVAP removed from human patients (n = 279) with suspected catheter-related infection. (DOCX) [file pone.0037281.s008.docx]

**Supplementary Table S1.** Microbiological analysis of TIVAP removed from human patients (n=279) with suspected catheter-related infection.

| **Microorganisms** | | | **n (%)** |
| --- | --- | --- | --- |
| Gram-positive cocci | | | 194 (66.4) |
|  | *Staphylococcus aureus* | | 91 |
|  | Coagulase-negative staphylococci | |  |
|  |  | *S. epidermidis* | 54 |
|  |  | *S. haemolyticus* | 5 |
|  |  | *S. hominis* | 4 |
|  |  | *S. lugdunensis* | 1 |
|  |  | Non characterized Co-NS | 34 |
|  | *Streptococcus pyogenes* | | 3 |
|  | *Enterococcus* spp. | | 2 |
| *Enterobacteriaceae* | | | 48 (16.4) |
|  | *Escherichia coli* | | 17 |
|  | *Enterobacter cloacae* | | 14 |
|  | *Klebsiella pneumoniae* | | 7 |
|  | *Enterobacter aerogenes* | | 2 |
|  | *Citrobacter freundii* | | 2 |
|  | *Serratia marcescens* | | 2 |
|  | *Klebsiella oxytoca* | | 2 |
|  | *Proteus mirabilis* | | 1 |
|  | *Citrobacter koseri* | | 1 |
| Aerobic Gram-negative rods | | | 36 (12.3) |
|  | *Pseudomonas aeruginosa* | | 27 |
|  | *Stenotrophomonas maltophilia* | | 5 |
|  | *Acinetobacter baumanii* | | 3 |
|  | *Pseudomonas* spp. | | 1 |
| Yeasts | | | 9 (3.1) |
|  | *Candida albicans* | | 5 |
|  | *Candida* spp. *(*non *albicans)* | | 4 |
| Other | | | 5 (2) |
| Total | | | 292 |

**NOTE.** Other: *Corynebacterium* spp. (n=4), *Bacillus* spp. (n=1)
