# Supplementary material for: A Rat Model of Central Venous Catheter to Study Establishment of Long-Term Bacterial Biofilm and Related Acute and Chronic Infections
Source: PLoS One. 2012 May 16;7(5):e37281. doi: 10.1371/journal.pone.0037281 (PMC3353920; doi:10.1371/journal.pone.0037281)
Supplement: Table S2 — In vivo relation between ROI (p/s/cm2/sr) and CFU/ml in port of implanted TIVAP at 10 dpi. (DOCX) [file pone.0037281.s009.docx]

**Supplementary Table S2**. *In vivo* relation between ROI (p/s/cm^2^/sr) and CFU/ml in port of implanted TIVAP at 10 dpi.

| **Strain** | **CFU/ml +/- SD (port)** | **ROI (p/s/cm^2^/sr) +/- SD (port)** |
| --- | --- | --- |
| ***E. coli*** | 4.64 x 10^7^ +/- 4.68 x10^6^ | 3.8 x10^4^ +/- 3.43 x 10^4^ |
| ***P. aeruginosa*** | 4.88 x 10^8^ +/- 3.42 x 10^8^ | 1.47 x 10^4^ +/-8.82 x 10^3^ |
| ***S. aureus*** | 2.07 x10^8^ +/- 1.68 x 10^8^ | 8.51 x 10^3^ +/- 1.09 x10^4^ |

number of rats is *E. coli* n=5, *P. aeruginosa* n=5 and *S. aureus* n=4
